# Stereoisomers of Saponins in *Panax notoginseng* (Sanqi): A Review

**DOI:** 10.3389/fphar.2018.00188

**Published:** 2018-03-13

**Authors:** Ming Peng, Ya X. Yi, Tong Zhang, Yue Ding, Jian Le

**Affiliations:** ^1^School of Pharmacy, Shanghai University of Traditional Chinese Medicine, Shanghai, China; ^2^Department of Chemistry, Shanghai Institute for Food and Drug Control, Shanghai, China; ^3^Experiment Center for Teaching and Learning, Shanghai University of Traditional Chinese Medicine, Shanghai, China; ^4^Shanghai Institute of Pharmaceutical Industry, China State Institute of Pharmaceutical Industry, Shanghai, China

**Keywords:** *Panax notoginseng*, processing, ginsenosides, saponins, stereoisomer, stereospecific, chiral

## Abstract

*Panax notoginseng* (Sanqi), a traditional Chinese medical drug which has been applied to medical use for over four centuries, contains high content of dammarane-type tetracyclic triterpenoid saponins. A number of stereoisomeric dammarane-type saponins exist in this precious herb, and some are particularly regarded as “biomarkers” in processed notoginseng. Contemporary researches have indicated that some saponin stereoisomers may show stereospecific pharmacological activities, such as anti-tumor, antioxidative, anti-photoaging, anti-inflammatory, antidiabetic, and neuro-protective activities, as well as stereoselective effects on ion channel current regulation, cardiovascular system, and immune system. The current review provides a comprehensive overview of chemical compositions of raw and processed *P. notoginseng* with a particular emphasis on saponin stereoisomers. Besides, the pharmacological and pharmacokinetic researches, as well as determination and biotechnological preparation methods of stereoisomeric saponins in notoginseng are discussed extensively.

## Introduction

Notoginseng, the root of *Panax notoginseng* (Burk.) F. H. Chen (*P. notoginseng*), also called Sanqi or Sanchi, is a precious traditional Chinese medical drug which has a history of medical use for over 400 years. The main active components of notoginseng include saponins, dencichine, flavonoids, polysaccharides and fatty acids, etc. (Wang et al., 2006, 2016). Although some non-saponin constitutents exhibit hemostasis, neuroprotective and immunity activities (Huang et al., 2014; Jia et al., 2014), a majority of the pharmacological functions of notoginseng are basically attributed to its saponin components (Shi et al., 2016). Contemporary research have discovered that *Panax notoginseng* saponins (PNS) can be effective in the treatment of cardiovascular diseases (Yang X. et al., 2014), such as atherosclerosis (Liu G. et al., 2009), hypertension (Pan et al., 2012), myocardial ischemia (Han et al., 2013), and aortic intimal hyperplasia (Wu et al., 2010), etc. Moreover, PNS also possesses the biological activities of anti-cancer (Yang et al., 2016), anti-hyperlipoidemia (Xia et al., 2011), anti-hyperglycemia (Yang et al., 2010), anti-inflammatory response (Rhule et al., 2008), anti-depression (Xiang et al., 2011), neuroprotective effect (Luo et al., 2010), antioxidative effect (Zhou et al., 2014), and bone formation stimulation activity (Chen et al., 2012), etc.

**FIGURE 1:**
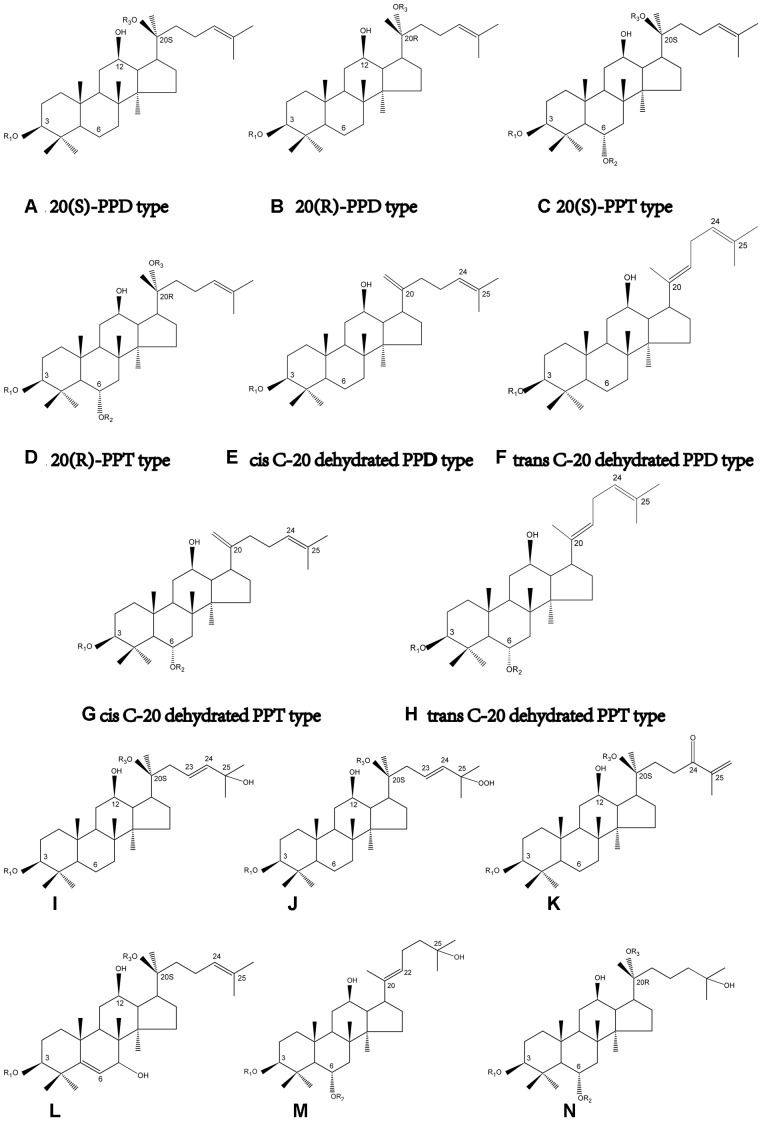
Structure skeletons of saponins existed in raw and processed *P. notoginseng*. The different substituents represented by R1, R2, and R3 in structures of **(A–N)** have been elaborated in **Table 1**.

Unlike its close relatives, i.e., *Panax ginseng* and *Panax quinque foilium, P. notoginseng* possesses dammarane-type tetracyclic triterpenoid saponins exclusively (Wang et al., 2006), and the total amount of dammarane-type saponins in *P. notoginseng* is evidently greater than that in the other two species (Wan et al., 2007). Dammarane-type saponins can essentially be divided into two groups: protopanaxadiol (PPD) and protopanaxatriol (PPT) type. Both of PPD and PPT aglycons bear chiral carbons in their structure skeletons, which lead to the stereoisomerism of PPD and PPT saponins (**Figures 1A–D**). Interestingly, although stereoisomeric compounds are widely distributed in herbal medical drugs, the chemical and pharmacological difference in stereoisomers in phytochemistry has not been extensively studied until recently, and the research targets include triterpenoid saponins (Nose et al., 1994), flavanones (Ren et al., 2007), Schisandrin B (Luk et al., 2008), alkaloids (Krizevski et al., 2010), fatty acids (Nagai et al., 2010), and pyranocoumarins (Song et al., 2014b).

The investigation on C-20 stereoisomerism of dammarane-type saponins has aroused the interest of researchers in recent years. Generally speaking, stereoisomerism in phytochemistry refers to optical and geometric isomerism, respectively. Not surprisingly, raw notoginseng is discovered to contain 20(S)-optical isomer of saponins basically, such as 20(S)-notoginsengoside R_2_, 20(S)-ginsenoside Rg_2_ and 20(S)-ginsenoside Rh_1_. However, it has been demonstrated that the processing procedures of *P. notoginseng*, e.g., steaming, heating, frying, etc., may lead to the cleavage of glycosyl linkage bonds and the dehydration at C-20. During the processes, some originally existed saponins in raw notoginseng would gradually be deglycosylated at C-20 to optical saponin epimers, such as 20(S)-/20(R)-ginsenoside Rg_3_ and 20(S)-/20(R)-ginsenoside Rh_2_, as well as be dehydrated at C-20 to geometric isomers, i.e., *cis-trans* isomers, such as ginsenoside RK_3_ and Rh_4_, RK_1_ and Rg_5_, RK_2_ and Rh_3_, etc. (**Figures 1E–H**) (Wang et al., 2012). Based on previously published studies, some secondary saponins are of high biological activities compared with the original ones in raw *P. notoginseng*. So far, literatures have indicated that saponin stereoisomers may exhibit significant difference in the following aspects: (1) physical and chemical properties; (2) methods of isolation, analysis and preparation; (3) biological, pharmacological, pharmacodynamic, and clinical evaluations; (4) key parameters of pharmacokinetics. However, the biotechnological difference of the stereoisomers of saponins in notoginseng has not been extensively reviewed so far. This article compiles a review of chemical compositions of raw and processed notoginseng with a particular emphasis on the stereoisomeric saponins. Besides, the determination, preparation methods, and the pharmacological and pharmacokinetic researches of those saponin stereoisomers are also elaborated.

## Chemistry

### Dammarane-Type Saponins in Raw *P. notoginseng*

So far extensive researches have been performed on the identification and analysis of saponins in raw *P. notoginseng*. The nomenclatures of the saponins isolated from *P. notoginseng* are generally ginsenoside and notoginsenoside. One distinguishing feature of notoginseng is that all of the saponins can be classified into PPD, PPT or their derivative types. However, the saponins profiles in different medicinal parts, e.g., root, rhizome, stem, flower, and leaf, etc., of notoginseng are of huge difference (Wan et al., 2012). The flower buds of notoginseng contain PPD type saponins predominantly. Nevertheless, both PPD and PPT type saponins are plentiful in roots (Yang et al., 2013). HPLC-ELSD and LC-QTOF-MS analysis also revealed the fact that the majority saponins in notoginseng leaves are of PPD types (Wang et al., 2015). An UPLC-ESI-MS method combined with principal component analysis (PCA) tentatively gave critical marker compounds in different part of notoginseng for the first time. The marker compounds assigned are listed below: (1) in roots: ginsenoside Rb_1_ and Rg_1_, notoginsenoside A and B, and 20-*O*-glucoginsenoside-Rf; (2) in flower buds: notoginsenoside Q, S and Fc, ginsenoside Rb_2_, Rb_3_, and F_2_; (3) in rhizomes: ginsenoside Re, Rf, Rg_2_, Rc, Rd, Rh_2_ notoginsenoside R_1_, R_4_, Fa and H, and malonyl-ginsenoside-Rb_1_ (Dan et al., 2008). It is noteworthy that although most of the naturally existed optical saponins are 20(S)-epimers such as 20(S)-Rg_2_ and 20(S)-Rh_1_, there are still investigations indicating 20(R)-Rg_3_ occurred in raw notoginseng (Dan et al., 2008; Qi et al., 2012; Sakah et al., 2013). 20(S)- and 20(R)-Rg_3_ are the only pair of C-20 stereoisomers reported in raw *P. notoginseng*. However, based on our results (Peng et al., 2016b), only 20(S)-form stereoisomeric saponins could be found in raw *P. notoginseng*. Take 20–80 head raw herbs obtained from Yunnan province as an example, 20(S)-notoginsenoside R_1_, 20(S)-Rg_2_ and 20(S)-Rh_1_ were found, and the amount was in the range of 1.0–1.9, 0.5–1.5, and 0.3–2.1 mg/g, respectively. The structures and locations of saponins existed in raw and processed *P. notoginseng* are summarized in **Figure 1** and **Tables 1A**,**B**.

**Table 1A:** Saponins existed in raw *Panax notoginseng*.

Structure skeleton	Saponin name	R1	R2	R3	Plant parts	Reference
A	Ginsenoside F_2_	-Glc	/	-Glc	Roots, rhizome, flower	Dan et al., 2008; Wan et al., 2012
A	Gypenoside XVII	-Glc	/	-Glc^6^-^1^Glc	Roots	Sakah et al., 2013
A	Notoginsenoside O	-Glc	/	-Glc^6^-^1^Xyl^3^-^1^Xyl	Leaf, flower	Wan et al., 2012
A	Notoginsenoside P	-Glc	/	-Glc^6^-^1^Xyl^4^-^1^Xyl	Leaf, flower	Wan et al., 2012
A	Ginsenoside Rh_2_^∗^	-Glc	/	-H	Rhizome	Dan et al., 2008
A	Ginsenoside Rd	-Glc^2^-^1^Glc	/	-Glc	Roots, rhizome, seed, stem, leaf, flower	Wan et al., 2012; Zhu et al., 2014
A	Ginsenoside Rc	-Glc^2^-^1^Glc	/	-Glc^6^-^1^Ara(f)	Roots, rhizome, seed, stem, leaf, flower	Wan et al., 2012
A	Ginsenoside Ra_2_	-Glc^2^-^1^Glc	/	Glc^6^-^1^Ara(f)^2^-^1^Xyl	Flower	Wan et al., 2012
A	Ginsenoside Rb_2_	-Glc^2^-^1^Glc	/	-Glc^6^-^1^Ara(p)	Roots, rhizome, seed, stem, leaf, flower	Wan et al., 2012; Zhu et al., 2014
A	Ginsenoside Ra_1_	-Glc^2^-^1^Glc	/	-Glc^6^-^1^Ara(p)^4^-^1^Xyl	Flower	Wan et al., 2012
A	Ginsenoside Rb_1_	-Glc^2^-^1^Glc	/	-Glc^6^-^1^Glc	Roots, rhizome, seed, stem, leaf, flower	Wan et al., 2012
A	Ginsenoside Ra_3_	-Glc^2^-^1^Glc	/	-Glc^6^-^1^Xyl^3^-^1^Xyl	Roots, rhizome	Wan et al., 2012; Zhu et al., 2014
A	Ginsenoside Rb_3_	-Glc^2^-^1^Glc	/	-Glc^6^-^1^Xyl	Roots, rhizome, seed, stem, leaf, flower	Wan et al., 2012
A	20(S)-Ginsenoside Rg_3_	-Glc^2^-^1^Glc	/	-H	Roots, rhizome, stem, leaf, flower	Wan et al., 2012
A	Notoginsenoside K	-Glc^6^-^1^Glc	/	-Glc	Roots, rhizome, seed, stem, leaf, flower	Wan et al., 2012
A	Notoginsenoside R_4_	-Glc^2^-^1^Glc	/	-Glc^6^-^1^Glc^6^-^1^Xyl	Roots, rhizome	Wan et al., 2012
A	Malonyl-ginsenoside Rb_1_	-Glc^2^-^1^Glc^6^-Malonoyl	/	-Glc^6^-^1^Glc	Roots, rhizome	Zhu et al., 2014
A	Notoginsenoside S	-Glc^2^-^1^Glc^2^-^1^Xyl	/	-Glc^6^-^1^Ara^5^-^1^Xyl	Flower	Wan et al., 2012; Yang et al., 2013
A	Notoginsenoside Fa	-Glc^2^-^1^Glc^2^-^1^Xyl	/	-Glc^6^-^1^Glc	Roots, rhizome, stem, leaf, flower	Wan et al., 2012; Zhu et al., 2014
A	Notoginsenoside Fc	-Glc^2^-^1^Glc^2^-^1^Xyl	/	-Glc^6^-^1^Xyl	Flower	Wan et al., 2012
A	Notoginsenoside Q	-Glc^2^-^1^Glc^2^-^1^Xyl	/	-Glc^6^-^1^Xyl^4^-^1^Xyl	Flower	Wan et al., 2012; Yang et al., 2013
A	Notoginsenoside L	-Glc^2^-^1^Xyl	/	-Glc^6^-^1^Glc	Roots, rhizome, seed, stem, leaf, flower	Wan et al., 2012
B	20(R)-Ginsenoside Rg_3_	-Glc^2^-^1^Glc	/	-H	Roots, rhizome	Qi et al., 2012; Sakah et al., 2013
C	Ginsenoside F_1_	-H	/	-Glc	Roots, rhizome	Wan et al., 2012
C	Chikusetsusaponin L_5_	-H	/	-Glc^6^-^1^Ara(p)^4^-^1^Xyl	Roots, rhizome, stem, leaf, flower	Wan et al., 2012
C	Notoginsenoside U	-H	/	-Glc^6^-^1^Glc	Roots	Sun et al., 2006
C	Ginsenoside Rg_1_^∗^	-H	-Glc	-Glc	Roots, rhizome, seed, stem, leaf, flower	Wan et al., 2012; Zhu et al., 2014
C	Notoginsenoside R_3_	-H	-Glc	-Glc^6^-^1^Glc	Roots, rhizome	Wan et al., 2012
C	Notoginsenoside R_6_	-H	-Glc	-Glc^6^-^1^αGlc	Roots, rhizome	Wan et al., 2012
C	Ginsenoside Rh_1_^∗^	-H	-Glc	-H	Roots, rhizome	Wan et al., 2012
C	20-*O*-Glucoginsenoside Rf	-H	-Glc^2^-^1^Glc	-Glc	Roots, rhizome	Wan et al., 2012
C	Ginsenoside Rf	-H	-Glc^2^-^1^Glc	-H	Roots, rhizome, stem	Wan et al., 2012
C	Notoginsenoside N	-H	-Glc^4^-^1^Glc	-Glc	Roots, rhizome	Wan et al., 2012
C	Ginsenoside Re	-H	-Glc^2^-^1^Rha	-Glc	Roots, rhizome	Wan et al., 2012; Zhu et al., 2014
C	Ginsenoside Rg_2_^∗^	-H	-Glc^2^-^1^Rha	-H	Roots, rhizome, seed, stem	Wan et al., 2012; Yang et al., 2013; Zhu et al., 2014
C	Notoginsenoside R_1_	-H	-Glc^2^-^1^Xyl	-Glc	Roots, rhizome, seed, stem, leaf, flower	Wan et al., 2012; Zhu et al., 2014
C	Notoginsenoside R_2_	-H	-Glc^2^-^1^Xyl	-H	Roots, rhizome	Wan et al., 2012
I	Notoginsenoside E	-Glc^2^-^1^Glc	/	-Glc	Roots, rhizome	Wan et al., 2012
I	Notoginsenoside A	-Glc^2^-^1^Glc	/	-Glc^6^-^1^Glc	Roots, rhizome	Wan et al., 2012
I	Yesanchinoside H	-Glc^2^-^1^Glc	/	-Glc^6^-^1^Xyl	Roots, rhizome	Wan et al., 2012
J	Notoginsenoside I	-Glc^2^-^1^Glc	/	-Glc^6^-^1^Glc	Roots, rhizome	Wan et al., 2012
K	Notoginsenoside B	-Glc^2^-^1^Glc	/	-Glc^6^-^1^Glc	Roots	Dan et al., 2008
L	Notoginsenoside G	-Glc^2^-^1^Glc	/	-Glc	Roots, rhizome	Wan et al., 2012


*^∗^Optical conformation not defined.*

**Table 1B:** Saponins produced in processed *Panax notoginseng*.

Structure skeleton	Saponin name	R1	R2	R3	Reference
A	20(S)-Ginsenoside Rh_2_	-Glc	/	-H	Chan et al., 2007
A	20(S)-Ginsenoside Rg_3_	-Glc^2^-^1^Glc	/	-H	Lau et al., 2004; Wang et al., 2012
A	20(S)-Ginsenoside Rs_3_	-Glc^2^-^1^Glc^6^-Ac	/	-H	Chan et al., 2007
A	6”-*O*-Acetylginsenoside Rg_3_	-Glc^2^-^1^Glc^6^-Ac	/	-Glc	Liao et al., 2008
B	20(R)-Ginsenoside Rh_2_	-Glc	/	-H	Chan et al., 2007
B	20(R)-Ginsenoside Rg_3_	-Glc^2^-^1^Glc	/	-H	Lau et al., 2004; Wang et al., 2012
C	20(S)-Ginsenoside Rh_1_	-H	-Glc	-H	Wang et al., 2012
C	Koryoginsenoside-R_1_	-H	-Glc^6^-(*E*)-2-Butenoyl	-Glc	Liao et al., 2008
C	Yesanchinoside D	-H	-Glc^6^-Ac	-Glc	Liao et al., 2008
C	20(S)-Ginsenoside Rg_2_	-H	-Glc^2^-^1^Rha	-H	Chan et al., 2007
C	20(S)-Protopanaxatriol	-H	-H	-H	Liao et al., 2008
D	20(R)-Ginsenoside Rh_1_	-H	-Glc	-H	Lau et al., 2004; Wang et al., 2012
D	20(R)-Ginsenoside Rg_2_	-H	-Glc^2^-^1^Rha	-H	Chan et al., 2007
D	20(R)-Protopanaxatriol	-H	-H	-H	Liao et al., 2008
E	Gingsenoside RK_1_	-Glc^2^-^1^Glc	/	/	Lau et al., 2004; Wang et al., 2012
E	Gingsenoside Rs_5_	-Glc^2^-^1^Glc^6^-Ac	/	/	Chan et al., 2007
F	Ginsenoside Rg_5_	-Glc^2^-^1^Glc	/	/	Lau et al., 2004; Wang et al., 2012
F	Ginsenoside Rs_4_	-Glc^2^-^1^Glc^6^-Ac	/	/	Chan et al., 2007
G	Gingsenoside RK_3_	-H	-Glc	/	Lau et al., 2004; Wang et al., 2012
G	Gingsenoside Rg_6_	-H	-Glc^2^-^1^Rha	/	Chan et al., 2007
G	3β,6α,12β-Trihydroxydammar-20(21),24-diene	-H	-H	/	Liao et al., 2008
H	Ginsenoside Rh_4_	-H	-Glc	/	Lau et al., 2004; Wang et al., 2012
H	Ginsenoside F_4_	-H	-Glc^2^-^1^Rha	/	Chan et al., 2007
M	Sanchinoside B_1_	-H	-Glc	/	Liao et al., 2008
N	25-Hydroxy-20(R)-Rh_1_	-H	-Glc	-H	Liao et al., 2008


*Structure skeletons and the positions of R_*1*_, R_*2*_, and R_*3*_ please refer to **Figure 1**; Glc, β-*D*-glucopyranosyl; Rha, rhamnose; Ara(p), α-*L*-Arabinose in pyranose form; Ara(f), α–*L*-Arabinose in furanose form; Xyl, xylose.*

### The Processing of *P. notoginseng*

The processing of traditional Chinese medical drug is a national heritage of a Chinese medicinal culture. The purpose of processing can be classified into four points: (1) eliminating or alleviating the toxicity and side effects; (2) changing the medicinal properties; (3) being convenient for formulation and storage; (4) cleaning the medical drug. In the case of *P. notoginseng*, the medicinal properties are altered upon the processing, i.e., baking, steaming, boiling and frying according to literatures and conventional Chinese methods (Lau et al., 2003, 2004; Chan et al., 2007; Sun et al., 2010; Toh et al., 2010; Wang et al., 2012). Among those processing procedures, steaming is most frequently used and the procedure of steaming at 100°C for 3 h has been set as the provincial standard for processed notoginseng powder in Yunnan, China, since April 1, 2013. Temperatures of 100°C and 120°C are practically used in the processing according to published literatures.

### Saponins Produced in Processed *P. notoginseng*

With the processing of notoginseng, some originally existed saponins will be degraded, accompanied by the generation of secondary saponins, including quite a number of saponin stereoisomers.

A series of measurements of HPLC-DAD, LC-MS/MS, LC-QTOFMS and NMR, etc., have been performed on the identification of saponins. Although some of the newly generated saponins are regarded as biomarkers for processed notoginseng, not much emphasis has been put on the presence of stereoismeric saponins so far, which is absolutely a very attractive topic.

The research group of Lau et al. (2003) and Lau et al. (2004) is by far the first team to investigate the difference of whole chromatograms of raw and steamed *P. notoginseng*. Results showed that with the extending of steaming time from 0 to 9 h in an autoclave at 120°C, the chromatograms of steamed notoginseng exhibited greater difference with that of raw materials. Except ginsenoside Rc, all the rest of known saponins, i.e., notoginsenoside R_1_, ginsenoside Rg_1_, Re, Rb_1_ and Rd were dramatically degraded. The newly occurred saponins include ginsenoside 20(R)-Rh_1_, Rk_3_, Rh_4_, 20(S)-/20(R)-Rg_3_, Rk_1_ and Rg_5_, which are all stereoisomeric saponins without exception.

The increasing content of 4 pairs of stereoisomeric saponins, i.e., 20(S)-/20(R)-Rh_1_, 20(S)-/20(R)-Rg_3_, Rk_3_ and Rh_4_, and RK_1_ and Rg_5_, upon the extending of steaming and baking of notoginseng were reported (Wang et al., 2012). Two *trans* C-20 dehydrated dammarane-type saponins, i.e., Rh_4_ and Rg_5_, were found to be of significantly higher contents compared with other 6 stereoisomeric saponins in processed notoginsengs. The contents of the two saponins reached 1% (w/w) after the dry notoginseng was steamed in an autoclave at 120°C for 8 h.

Chan et al. (2007) firstly introduced the name “biomarker” into steamed notoginseng. Here, the “biomarker” means the compounds only existed in steamed notoginseng or those of quite high content in steamed notoginseng while extremely low content in raw materials. Here, the biomarkers tentatively given in steamed notoginseng include 8 pairs of stereoisomeric saponins, i.e., ginsenoside 20(S)-/20(R)-Rg_2_, 20(S)-/20(R)-Rh_1_, 20(S)-/20(R)-Rg_3_, 20(S)-/20(R)-Rs_3_, Rg_6_ and F_4_, Rk_3_ and Rh_4_, RK_1_ and Rg_5_, and Rs_5_ and Rs_4_. Besides, ginsenoside 20(S)-/20(R)-Rh_2_ were also detected in steamed notoginseng, with quiet minor concentrations though. The concept of biomarker could be successfully used to differentiate raw and processed notoginseng.

Statically data has indicated that the content of 20(S)-Rg_3_ in steamed notoginseng reaches its maximum after a 6 h steaming in an autoclave at 120°C, and the prolongation of steaming time would not significantly increase the content of 20(S)-Rg_3_ anymore. However, in the case of 20(S)-Rh_2_, the steaming time should be extended to 24 h for the achievement of its maximum content (Toh et al., 2010). The structures of saponins newly generated in raw *P. notoginseng* are also summarized in **Figure 1** and **Table 1**. In summary, there are one pair C-20 stereoisomers, i.e., 20(S)-/20(R)-Rg_3_, in raw notoginseng, and ten pairs of C-20 stereoisomers, including 20(S)-/20(R)-Rg_3_, 20(S)-/20(R)-Rh_2_, 20(S)-/20(R)-Rh_1_, 20(S)-/20(R)-Rg_2_, 20(S)-/20(R)-Rs_3_, RK_2_/Rh_3_, Rg_6_/F_4_, Rk_3_/Rh_4_, RK_1_/Rg_5_, and Rs_5_/Rs_4_ reported in processed *P. notoginseng*.

**FIGURE 2:**
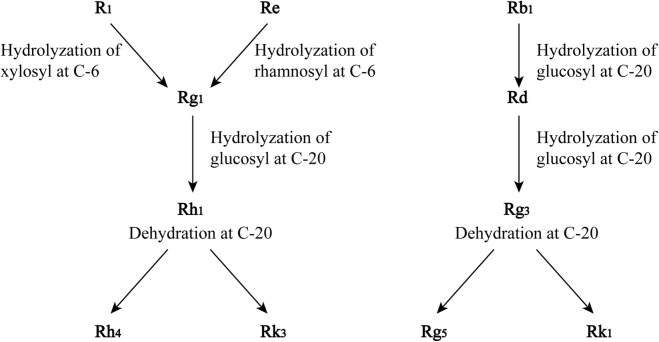
Proposed transformation of saponins in the process of steaming of notoginseng. Adopted from Wang et al. (2012).

The mechanism of saponin transformations into sapogenins/prosapogenins in the steaming process has been extrapolated from the chemical structures of saponins, especially the changes in their sugar moieties. Under the steaming condition, the hydrolysis of the xylosyl residue attached to C-6 of notoginsenoside R_1_ and the hydrolysis of the rhamnosyl residue at C-6 of ginsenoside Re form ginsenoside Rg_1_. Ginsenoside Rb_1_ is hydrolyzed at the glucosyl residue at C-20 to yield ginsenoside Rd. Rg_1_ and Rd are likely to be the parent compounds of newly formed saponins. The further hydrolysis of the glucosyl residue at C-20 of Rg_1_ yields Rh_1_ which then forms Rh_4_ and Rk_3_ through dehydration at C-20. Similarly, the hydrolysation of the glucosyl residue at C-20 of Rd produces Rg_3_, and dehydration of Rg_3_ at C-20 yields Rg_5_ and Rk_1_, which are shown in **Figure 2** (Wang et al., 2012).

### Determination Strategy for Saponin Stereoisomers

As we all know, 20(S)- and 20(R)-ginsenoside epimers bear very similar molecular structures, and their fragment ions in mass spectra are the same. Recently, Qi et al. (2012) developed a LC-TOF-MS method to differentiate 20(S)- from 20(R)-ginsenoside epimers based on the mass spectra in positive ion mode. In their study, a slight difference in the peak ratio of [M-2H_2_O+H]^+^ to [M-H_2_O+H]^+^ has been found in these two epimers. Since the steric hindrance of 20(R)-epimer is greater than that of 20(S)-epimer during the dehydration at the C-20 hydroxyl group, the [M-2H_2_O+H]^+^/[M-H_2_O+H]^+^ ratio is higher for 20(S)-epimer (1:1) compared with 20(R)-epimer (0.7:1). As the chemical features of 20(S)- and 20(R)-epimers are very close, the retention times of these two epimers might be overlapped according to the separation chromatographic columns. This identification method is of particular importance to distinguish 20(S)- from 20(R)-ginsenoside epimers.

One-dimensional and two-dimensional Nuclear Magnetic Resonance (NMR) spectroscopy is a very useful technique to distinguish the structures of stereoisomers of saponins. Specifically, ^13^C-NMR spectral data can provide information for structural elucidation of ginsenoside isomers. The chemical shifts of the characteristic peaks of 20(S)- and 20(R)- ginsenosides provide information for the identification of stereoisomers. In particular, the changes in chemical shift between 20(S)- and 20(R)-epimers at C-17, C-21 and C-22 in the ^13^C-NMR spectra are approximately Δδ (δ*_S_*– δ*_R_*) +4.1 ± 0.1, +4.3 ± 0.1, and –7.4 ± 0.0 ppm (**Table 2**) (Yang H. et al., 2014).

**Table 2:** ^13^C-NMR spectroscopicdata for compounds 20(S)-/(R)-Rg_3_, 20(S)-/(R)-Rh_2_, 20(S-)/(R)-Rh_1_, 20(S)-/(R)-Rg_2_ in pyridine-d5.

No.	20(S)-Rg_3_^a^	20(R)-Rg_3_^a^	20(S)-Rh_2_^b^	20(R)-Rh_2_^b^	No.	20(S)-Rh_1_^a^	20(R)-Rh_1_^a^	20(S)-Rg_2_^a^	20(R)-Rg_2_^b^

δ _c_ multiplicity					δ _c_ multiplicity				
1	39.1 t	39.1 t	39.1 t	39.1 t	1	39.3 t	39.3 t	39.5 t	39.6 t
2	26.7 t	26.6 t	26.7 t	26.6 t	2	27.8 t	27.9 t	27.7 t	27.7 t
3	88.9 d	88.9 d	88.7 d	88.7 d	3	78.5 d	78.5 d	78.3 d	78.5 d
4	39.6 s	39.6 s	39.6 s	39.6 s	4	40.3 s	40.3 s	41.1 s	39.9 s
5	56.3 d	56.3 d	56.3 d	56.3 d	5	61.4 d	61.4 d	60.7 d	60.8 d
6	18.4 t	18.4 t	18.4 t	18.4 t	6	80.0 d	80.0 d	74.2 d	74.3 d
7	35.1 t	35.1 t	35.1 t	35.1 t	7	45.2 t	45.1 t	46.0 t	46.0 t
8	39.9 s	40.0 s	40.0 s	40.0 s	8	41.0 s	41.1 s	41.1 s	41.1 s
9	50.3 d	50.3 d	50.3 d	50.3 d	9	50.1 d	50.1 d	49.7 d	49.7 d
10	36.8 s	36.9 s	36.9 s	36.9 s	10	39.6 s	39.6 s	39.9 s	39.9 s
11	31.3 t	31.4 t	31.3 t	31.4 t	11	32.0 t	32.2 t	32.0 t	32.1 t
12	70.9 d	70.8 d	70.9 d	70.8 d	12	71.0 d	70.9 d	71.0 d	70.9 d
13	48.5 d	49.2 d	48.5 d	49.2 d	13	48.2 d	48.8 d	48.1 d	48.8 d
14	51.6 s	51.7 s	51.7 s	51.7 s	14	51.6 s	51.7 s	51.6 s	51.7 s
15	32.0 t	32.1 t	32.0 t	32.1 t	15	31.2 t	31.3 t	31.2 t	31.3 t
16	26.8 t	26.7 t	26.8 t	26.7 t	16	26.7 t	26.6 t	26.8 t	26.6 t
**17**	**54.7 d**	**50.6 d**	**54.7 d**	**50.6 d**	**17**	**54.7 d**	**50.5 d**	**54.6 d**	**50.5 d**
18	15.8 q	15.8 q	16.3 q	16.3 q	18	17.3 q	17.3 q	17.6 q	17.6 q
19	16.3 q	16.3 q	15.8 q	15.8 q	19	17.6 q	17.6 q	17.5 q	17.5 q
20	72.9 s	72.9 s	72.9 s	72.9 s	20	72.9 s	73.0 s	72.9 s	72.9 s
**21**	**27.0 q**	**22.7 q**	**27.0 q**	**22.7 q**	**21**	**26.9 q**	**22.7 q**	**27.0 q**	**22.7 q**
**22**	**35.8 t**	**43.2 t**	**35.8 t**	**43.2 t**	**22**	**35.8 t**	**43.2 t**	**35.7 t**	**43.2 t**
23	23.0 t	22.5 t	22.9 t	22.6 t	23	22.9 t	22.5 t	22.9 t	22.5 t
24	126.2 d	126.0 d	126.3 d	126.0 d	24	126.2 d	126.0 d	126.3 d	126.0 d
25	130.7 s	130.7 s	130.7 s	130.7 s	25	130.7 s	130.7 s	130.7 s	130.7 s
26	25.7 q	25.8 q	25.8 q	25.8 q	26	25.7 q	25.8 q	25.8 q	25.8 q
27	17.0 q	17.2 q	17.6 q	17.7 q	27	17.6 q	17.6 q	17.6 q	17.6 q
28	28.1 q	28.1 q	28.1 q	28.1 q	28	31.6 q	31.7 q	32.1 q	32.1 q
29	16.5 q	16.5 q	16.7 q	16.7 q	29	16.3 q	16.3 q	16.8 q	17.2 q
30	17.6 q	17.6 q	17.0 q	17.3 q	30	16.7 q	17.0 q	17.1 q	17.1 q

**3-*O*-β -D-Glucopyranosyl**					**6-*O*-α -C-Rhamnopyranosyl**				

1′	105.0 d	105.1 d	106.9 d	106.9 d	1′	106.0 d	106.0 d	101.9 d	101.9 d
2′	83.4 d	83.4 d	75.7 d	75.7 d	2′	75.4 d	75.4 d	79.4 d	79.4 d
3′	77.9 d	77.9 d	78.7 d	78.7 d	3′	79.6 d	79.6 d	78.5 d	78.5 d
4′	71.6 d	71.6 d	71.8 d	71.8 d	4′	71.8 d	71.8 d	72.4 d	72.4 d
5′	78.2 d	78.2 d	78.3 d	78.3 d	5′	78.1 d	78.1 d	78.3 d	78.3 d
6′	62.8 t	62.8 t	63.0 t	63.0 t	6′	63.0 t	63.0 t	63.0 t	63.1 t

**2′-*O*-β -D-Glucopyranosyl**					**2′-*O*-α -C-Rhamnopyranosyl**				

1″	106.0 d	106.0 d			1″			101.7 d	101.7 d
2″	77.1 d	77.1 d			2″			72.2 d	72.2 d
3″	78.3 d	78.3 d			3″			72.5 d	72.6 d
4″	71.6 d	71.6 d			4″			74.1 d	74.1 d
5″	78.0 d	78.1 d			5″			69.4 d	69.4 d
6″	62.7 t	62.7 t			6″			18.7 q	18.7 q
COCH_3_					COCH_3_				
COCH_3_					COCH_3_				


*Multiplicity of ^13^*C*-NMR data determined by distortionless enhancement by polarization transfer (DEPT) experiments. ^*a*^^13^*C*-NMR data of this compound was measured at 125 MHz and ^*b*^^13^*C*-NMR data of this compound was measured at 150 MHz. The bold values are the ^13^*c*-NMR spectra of C-17, C-21 and C-22 that particularly change in chemical shift between 20(S)- and 20(R)-epimers.*

*The letters “t, d, s, q” means triplet peak, double peak, single peak and quadruple peak respectively.*

High Performance Liquid Chromatography (HPLC) is the most frequently employed separation method for the analysis of saponins stereoisomers. HPLC can be combined with different detection techniques such as ultraviolet detector (UV), diode array detection (DAD), evaporative light scattering detector (ELSD), mass spectrometry detector (MS) and charged aerosol detector (CAD). There are two main difficulties that researchers come across in performing HPLC-UV or HPLC-DAD analysis of saponin stereoisomers. Firstly, owing to their very similar structures, saponin stereoisomers could not be baseline separated on a simple isocratic eluted chromatographic condition by an ordinary reversed-phase C18 column. A chromatographic method with gradient elution program is often necessary, and the parameters of the appropriate chromatographic column, e.g., packing material, particle size, length, etc., should be selected elaborately; Secondly, since saponins don’t possess aromatic groups, their UV absorptions are very low. Thus, only end absorption at around 200 nm could be chosen for UV or DAD detection. Therefore, ELSD, a universal and non-specific analytical detector, has been widely used in the detection of natural compounds including saponins. Firstly introduced in 2002, CAD, another universal detector for non-volatile and semi-volatile compounds, has been used in the detection of saponins in notoginseng. Moreover, MS and tandem MS detector are often used when the elucidation of saponin structures is needed, or when very high sensitivity for the detection of analytes is required. MS detector is particularly important in the detection of saponins in biological matrices.

Gradient elution of water and acetonitrile has been regarded as the most widely used chromatographic conditions for HPLC-UV or DAD separation of saponin stereoisomers, since acetonitrile has a fairly strong eluting power and its cut-off wavelength is lower than 200 nm, which makes it suitable for the detection at end absorption (Wang et al., 2008; Liu et al., 2010c; Dong et al., 2011; Park et al., 2013). In this case, UV wavelength is normally set at 203 nm for saponin determination. For ELSD and MS detectors, CH_3_COOH and HCOOH are usually added into mobile phases to assist a better volatilization of saponin analytes (Chan et al., 2007; Sun et al., 2009; Bae et al., 2013; Wan et al., 2013). Our group has developed a HPLC-CAD method to simultaneously separate 22 saponins existed in raw and processed notoginseng, in which 5 pairs of 20(S)- and 20(R)-ginsenoside epimers were separated (Peng et al., 2016b). In addition, using an appropriate column with small particle size can gain higher efficiency and better isolation. Ultra-performance liquid chromatography (UPLC), which utilizes silica particles of less than 2 μm, makes it possible to perform better separations in short periods of time (Chan et al., 2007; Park et al., 2013). The chromatographic conditions for simultaneous determination of stereoisomeric saponins are summarized in **Table 3**.

**Table 3:** Chromatographic conditions for simultaneous determination of stereoisomeric saponins.

Column	Mobile phase	Detector	Stereoisomeric saponins simultaneously separated	Reference
Zorbax Eclipse XDB-C18 (25 cm × 4.6 mm, 5 μm)	A: H_2_O, B: CH_3_CN; gradient elution	UV, 203 nm	20(S)-/20(R)-Rg_2_, 20(S)-/20(R)-Rh_1_, 20(S)-/20(R)-Rg_3_, 20(S)-/20(R)-Rh_2_, Rk_1_/Rg_5_, Rg_6_/F_4_, Rk_3_/Rh_4_, 20(S)-/20(R)-PPD	Dong et al., 2011
Discovery C18 (25 cm × 4.6 mm, 5 μm)	A: H_2_O, B: CH_3_CN; gradient elution	UV, 203 nm	20(S)-/20(R)-Rg_3_, 20(S)-/20(R)-Rh_2_, 20(S)-/20(R)-PPD	Liu et al., 2010c
ACQUITY BEH C18 (10 cm × 2.1 mm, 1.7 μm)	A: 0.001% H_3_PO_4_, B: 0.001% H_3_PO_4_ in CH_3_CN; gradient elution	DAD, 203 nm	20(S)-/20(R)-Rg_2_, 20(S)-/20(R)-Rg_3_, 20(S)-/20(R)-Rh_2_, Rk_1_/Rg_5_, Rg_6_/F_4_, Rk_3_/Rh_4_, RK_2_/Rh_3_	Park et al., 2013
Waters HSS C18 column (25 cm × 4.6 mm, 3.5 μm)	A: H_2_O, B: CH_3_CN; gradient elution	CAD; UV 203 nm	20(S)-/20(R)-Rg_2_, 20(S)-/20(R)-Rh_1_, 20(S)-/20(R)-Rg_3_, 20(S)-/20(R)-Rh_2_, 20(S)-/20(R)-PPD	Peng et al., 2016b
Zorbax Eclipse XDB-C18 (25 cm × 4.6 mm, 5 μm)	A: H_2_O, B: CH_3_CN; gradient elution	UV 203 nm	20(S)-/20(R)-Rg_2_, 20(S)-/20(R)-Rg_3_, 20(S)-/20(R)-Rs_3_, Rk_1_/Rg_5_, Rg_6_/F_4_, RS_4_/Rh_5_	Wang et al., 2008
Discovery C18 (25 cm × 4.6 mm, 5 μm)	A: CH_3_CN-H_2_O-5% CH_3_COOH (10:85:5), B: CH_3_CN-H_2_O (80:20); gradient elution	ELSD; probe temperature: 60°C; nebulizer flow: N_2_ 1.8 L/min	20(S)-/20(R)-Rg_3_, 20(S)-/20(R)-Rs_3_, Rg_6_/F_4_, Rk_3_/Rh_4_, Rk_1_/Rg_5_, RS_4_/Rh_5_	Sun et al., 2009
ACQUITY C18 (10 cm × 2.1 mm, 1.7 μm)	A: 0.001% HCOOH, B: 0.001% HCOOH in CH_3_CN; gradient elution	TOF MS, ES+ and ES- mode	20(S)-/20(R)-Rg_2_, 20(S)-/20(R)-Rh_1_, 20(S)-/20(R)-Rg_3_, 20(S)-/20(R)-Rs_3_, F_4_/Rg_6_, Rk_3_/Rh_4_, RK_1_/Rg_5_, Rs_5_/ Rs_4_	Chan et al., 2007
Zorbax Extend C18 (25 cm × 4.6 mm, 5 μm)	A: 0.001% HCOOH, B: 0.001% HCOOH in CH_3_CN; gradient elution	QTOF MS, ESI- mode	20(S)-/20(R)-Rg_2_, 20(S)-/20(R)-Rh_1_, 20(S)-/20(R)-Rg_3_, 20(S)-/20(R)-Rh_2_, F_4_/Rg_6_, RK_1_/Rg_5_	Wan et al., 2013
Acclaim RSLC C18 (15 cm × 2.1 mm, 2.2 μm)	A: CH_3_CN, B: 0.1% HCOOH; gradient elution	Tandem MS, ES- mode	20(S)-/20(R)-Rg_3_, 20(S)-/20(R)-Rh_2_	Bae et al., 2013


### Preparation of Saponin Stereoisomers

As far as we know, major ginsenosides, e.g., Rb_1_, Rb_2_, Rd, Re, and Rg_1_, occupy 90% of total saponins which exist naturally in raw herbs. However, minor saponins, e.g., Rg_3_, Rh_2_, Rg_2_, Rh_1_, and F_1_, usually exert stronger pharmacological activities such as anti-tumor, antidiabetic, anti-oxidative, and anti-aging effects over the glycosylated major saponins (Choi et al., 2011; Kim Y.J. et al., 2013). A variety of technologies has been performed to produce minor saponins, and the processes include mild acid hydrolysis, alkali treatment and microbial conversions (Bae et al., 2002; Cheng et al., 2008; Cui et al., 2016). Ginsenoside Rb_1_, Rb_2_, Rc and Rd can be converted into a mixture of 20(S)- and 20(R)-Rg_3_ by either mild acid treatment or heating (Park, 2004). Nevertheless, side-reactions such as epimerization, hydration and hydroxylation are the disadvantages of chemical hydrolysis. Moreover, the optical purification from the mixture of 20(S)- and 20(R)-ginsenoside mixture is a time-consuming task. Fortunately, microorganic and enzymatic hydrolysis from major saponins has been found to be stereoselective and regarded as an efficient technology to avoid those side-reactions mentioned above (Liu et al., 2010c). Another advantage of this method is that the 20(S)-epimer produced would not transform to their 20(R)-epimer during the course of the reaction. Aspergillus niger obtained from soil shows a strong and stereoselective ability to transform Rg_3_(S, R) into PPD(S, R) completely. Microbacterium sp. GS514 isolated and identified from soil of a ginseng field exhibits a strong capability to convert ginsenoside Rb_1_ to 20(S)-Rg_3_ (Cheng et al., 2008). A new ginsenoside-transforming-glucosidase (BglQM) from *Mucilaginibacter* sp. Strain QM49 efficiently transforms ginsenoside Re and Rg_1_ into 20(S)-Rg_2_ and 20(S)-Rh_1_, respectively (Cui et al., 2013b). Moreover, Du et al. (2014) reported a method to transform Re to 20(S)-Rg_2_ with the aid of Pseudonocardia sp. Gsoil 1536, which can produce high-purity 20(S)-Rg_2_ in a 100 g scale. Various saponin-hydrolyzing pathways to produce stereoisomeric saponins from major saponins by enzymatic hydrolysis are summarized in **Table 4** for practical application.

**Table 4:** Major saponins stereoisomers transformations by glycoside hydrolases.

Name of glycoside hydrolase	Microorganism	Saponin conversion pathway	Reference
GS514	*Microbacterium* sp.	Rb_1_→Rd→Rg_3_(S)	Cheng et al., 2008
Glycosidase	*Aspergillus niger*	Rf→Rh_1_(S)→PPT(S)	Liu et al., 2010b
		Rg_3_(S)→Rh_2_(S)→PPD(S); Rg_3_(R)→Rh_2_(R)→PPD(R)	Liu et al., 2010c
Ginsenosidase type IV	*Aspergillus* sp. 39 g strain	Rg_1_/Re→Rh_1_(S)→PPT(S); Rf(S)/Rg_2_(S)→Rh_1_(S)→PPT(S)	Wang et al., 2011
Ginsenosidase type III	*Terrabacter ginsenosidimutans* sp.	Rg_3_(S)→Rh_2_(S)→PPD(S)	Jin et al., 2012
BglSk	*Sanguibacter keddieii*	Rg_3_(S)→Rh_2_(S)→PPD; Re→Rg_2_(S); Rb_1_→Rd→Rg_3_(S)	Kim J.-K. et al., 2012
Bgp1	*Microbacterium esteraromaticum*	Rb_1_→Rd→Rg_3_(S)	Quan et al., 2012
BglAm	*Actinosynnema mirum*	Rd→F_2_→Rh_2_(S); Rg_3_(S)→Rh_2_(S)→PPD(S); Re→Rg_2_(S); Rg_1_→Rh_1_(S)→PPT(S)	Cui et al., 2013a
BglQM	*Mucilaginibacter* sp. strain QM49	Re→Rg_2_(S); Rg_1_→Rh_1_(S); Rb_1_→Rd→Rg_3_(S)	Cui et al., 2013b
BglPC28	*Pseudonocardia* sp. strain Gsoil 1536	Re→Rg_2_(S); Rg_1_→Rh_1_(S); Rb_3_→Rg_3_(S); Rb_1_→Rg_3_(S); Rd→Rg_3_(S)	Du et al., 2014


## Pharmacology

### Pharmacological Difference Between Raw and Processed *P. notoginseng*

In traditional Chinese medical applications, processed notoginseng is distinguished from raw notoginseng by the claim of its capability to “nourish” blood (Medicine SAoTC, 1996). So far, not many contemporary researches have been performed to focus on the pharmacological difference between these two types of herbs, however, some research reports have still revealed that processed notoginseng exhibit more potent pharmaceutical activities than raw notoginseng, such as anticancer, antiplatelet and anticoagulant, and platelet aggregation inhibition effects, etc.

Compared with raw *P. notoginseng*, steamed *P. notoginseng* is found to be more potent in antiplatelet and anticoagulant effects *in vitro*. Moreover, it also exhibites stronger platelet aggregation inhibition effects *ex vivo*, and the effects could be enhanced along with the prolongation of steaming duration (Lau et al., 2009). A colorimetric WST-1 assay has been performed to evaluate the anti-proliferative effects of raw and steamed *P. notoginseng* on three human liver cancer cells, i.e., SNU449, SNU182 and HepG2. Results have indicated that steamed *P. notoginseng* presents higher anti-proliferative effects against these three types of liver cancer cells. Besides, the anti-proliferative effects of 5 originally existed saponins in raw *P. notoginseng*, i.e., Rg_1_, Rb_1_, Rd, Re and R_1_, as well as 4 newly produced saponins in steamed *P. notoginseng*, i.e., Rh_2_, Rk_1_, Rk_3_ and 20(S)-Rg_3_ have also been assessed. Not surprisingly, Rk_3_, Rh_2_, Rk_1_ and 20(S)-Rg_3_ exhibits more anti-proliferative effects compared with the original saponins (Toh et al., 2011). Moreover, the extract of steamed notoginseng is effective in the inhibition of proliferation of SW-480 human colorectal cancer cells. Meanwhile, the comparison results of the proliferation effects between ginsenoside Rg_1_, Rb_1_ and Rg_3_ shows that the newly generated ginsenoside Rg_3_ is the most potent saponin on the antiproliferative activities on SW-480 human colorectal cancer cells (Sun et al., 2010). This anticancer effect of steamed *P. notoginseng* was further confirmed by MTS method and flow cytometry (Sun et al., 2011).

Apparently, the difference of compound basis between raw and processed *P. notoginseng* directly influences their pharmacological activities. With the prolongation of processing duration, quite a few secondary saponins which are not naturally occurred in raw herbs are gradually generated in *P. notoginseng*. What should be emphasized is that most of the newly occurred saponins are stereoisomers, which exhibit quite potent biological activities. That explains why processed *P. notoginseng* exhibit distinct pharmacological behaviors compared with raw herbs.

### Stereospecific Pharmacological Effects of 20(S)- and 20(R)-Rg_3_

Ginsenoside Rg_3_ is one of the most biologically potent saponins in *P. notoginseng*, which exerts a wide scope of pharmacological actions, e.g., anti-inflammatory (Park et al., 2012), anti-tumor (Kim Y.-J. et al., 2014), antioxidant (Wei et al., 2012b), anti-diabetic (Ju et al., 2012; Kim S.S. et al., 2014), and neuroprotective (Lee B. et al., 2013) activities. Extensive research have revealed that ginsenoside Rg_3_ displays a wide spectrum of anticancer activities in the treatment of colon, lung (Kim Y.-J. et al., 2014), breast (Kim B.-M. et al., 2014), hepatic (Lee J.Y. et al., 2013), pancreatic (Guo et al., 2014) cancer and melanoma (Shan et al., 2015), via the mechanism of promoting cancer cell apoptosis, as well as inhibiting cell proliferation, metastasis and invasion. In addition, ginsenoside Rg_3_ has also been demonstrated to be a beneficial supplement in the enhancement of the inhibitory effects on chemotherapy (Sun et al., 2016).

At the early stages of pharmacological researches on this ginsenoside, the optical conformation was not specified, and the mixture of 20(S)- and 20(R)-Rg_3_ epimers has often been studied since very little information was obtained on their stereoselective differences in pharmacological actions (Choi et al., 2011). However, recent investigations have revealed that this pair of ginsenoside Rg_3_ stereoisomers exhibits a diversity of stereoselective activities, such as relaxation of coronary artery contractions, regulation of ion channel, effects on immune system, as well as anti-tumor, neuro-protective, anti-oxidant and antidiabetic activities, based on the chiral center at C-20 in their molecular structures.

#### Effects on Cardiovascular System

Ginsenoside Rg_3_ has potent pharmacological activities in cardiovascular systems. Stereospecific activities of Rg_3_ epimers have been discovered on coronary artery relaxation, endothelial cells survival, and platelet anti-aggregation, etc.

Investigations on swine coronary artery have revealed that 20(S)-Rg_3_, but not 20(R)-Rg_3_, engenders an effective coronary artery relaxation induced by 25mM KCl, which is concentration dependent yet endothelium independent. Moreover, although both Rg_3_ epimers could induce a significant, concentration-dependent relaxation of coronary artery contractions in intact samples induced by 3 mM 5-HT, only 20(S)-Rg_3_ inhibits coronary artery contraction in endothelium-denuded arteries. The stereospecific effects are probably owing to the inhibition of L-type Ca^2+^ channel or the elevation of Ca^2+^ level in the cells induced by 5-HT receptors (Kim et al., 2006). 20(S)-Rg_3_ has been found to show anti-apoptotic activity in human endothelial cells through Akt-dependent inhibition of the mitochondrial apoptotic signaling pathway. 20(S)-Rg_3_ has been shown to be comparable to 20 ng/ml of vascular endothelial growth factor (VEFG) on the promotion ability of endothelial cells survival, however, 20(R)-Rg_3_ only exhibits very little activity (Min et al., 2006). Moreover, Lee et al. (2009) investigated the anti-platelet aggregation activities of Rg_3_ epimers, and results showed that 20(S)-Rg_3_ strongly inhibited arachidonic acid-induced platelet aggregation, while 20(R)-Rg_3_ inhibited collagen-induced platelet aggregation.

#### Anti-tumor Effects

Quite a few researches have indicated that both 20(S)- and 20(R)-Rg_3_ exhibit anti-tumor activities, which have received much attention in recent investigations. Nevertheless, it is hard to say which stereoisomer exhibit higher anti-tumor activities based on available literatures so far, as these two compounds may function differently in different cell lines.

20(S)-Rg_3_ stereospecifically induces angiogenesis by promoting human endothelial cells proliferation, migration and tube formation *in vitro*, and endothelial sprouting *ex vivo* at a micromolar concentration level (Kwok et al., 2012). It is identified to be the main active component with anticancer effects on human gastric cancer AGS cells, while 20(R)-Rg_3_ has no effect (Park et al., 2014). Moreover, 20(S)-Rg_3_ is also found to be effective in other cell lines, such as human leukemic U937 and HL-60 cells (Qiu et al., 2014), multiple myeloma U266 cells (Song et al., 2014a), human ovarian cancer HO-8910 (Wang et al., 2014) and A2780 cells (Park et al., 2016), and colon cancer HT-29 cells (Yuan et al., 2010).

However, 20(R)-Rg_3_ shows a stronger anti-tumor effect on the inhibition of H22 transplanted tumors growth, as well as a higher immunomodulatory activity on H22-bearing mice, compared with its 20(S)-epimer, which may be attributed to its ability to stimulate lymphocyte proliferation and elevate cytokine levels in tumor-bearing mice (Wu et al., 2014). Furthermore, the comparison of the effects between 20(S)- and 20(R)-Rg_3_ on epithelial-mesenchymal transition (EMT) reveal that 20(R)-Rg_3_, but not 20(S)-Rg_3_, markedly increases the expression of the epithelial marker *E*-cadherin and represses Snail upregulation and the expression of the mesenchymal marker vimentin during the initiation of the TGF-β1-induced EMT. 20(R)-Rg_3_ also inhibits the TGF-β1-induced increasing in cell migration, invasion, and anoikis resistance of A549 lung cancer cells (Kim Y.-J. et al., 2014).

#### Effects on Ion Channel Current

20(S)- and 20(R)-Rg_3_ epimers are reported to exhibit stereospecific actions in ion channels, including Ca^2+^, K^+^, and Na^+^ (Kang et al., 2005; Lee et al., 2007). Without exception, 20(S)-Rg_3_, but not 20(R)-Rg_3_, has been demonstrated to significantly inhibit these channel currents in a dose- and voltage-dependent manner. The ^1^H and ^13^C NMR chemical shifts of all hydroxyl protons and the conformation of the tertiary structures of 20(S)- and 20(R)-Rg_3_ epimers indicates that a more potent interaction might be occurred between 20(S)-Rg_3_ with the receptor sections in ion channels compared with 20(R)-Rg_3_, probably due to the deduction that the hydroxyl group of 20(S)-Rg_3_ on C-20 may be geometrically better disposed to the hydroxyl receptor region in those ion channels. Moreover, 20(S)-Rg_3_ is more efficient on the regulation of mutant 5-HT3A receptor channel activity (Lee et al., 2007). It can be inferred that 20(S)-Rg_3_ might be a useful agent for regulation of ion channel current, which could be used for the treatment of coronary artery and neuron system disease. For instance, the neuro-protection mechanism and antinociceptive effects on inflammation of 20(S)-Rg_3_ is via the mechanism of regulating channels and receptors (Park et al., 2012; Kang et al., 2013).

#### Effects on Immune System

Several studies have highlighted the stereospecific effects of ginsenoside Rg_3_ in immune system. Wei et al. (2012a) utilized ovalbumin (OVA) as a model to assess the adjuvant effects of 20(S)-/20(R)-Rg_3_ epimers. Results showed that 20(R)-Rg_3_ exhibit higher adjuvant effects on OVA induced immune system in mice. Another study has compared the effects of these two epimers on the growth of hepatocellular carcinoma H22 transplanted tumors and the immune function of H22-bearing mice. And 20(R)-Rg_3_ has been found to be stereo-specifically inhibiting H22 tumor growth *in vivo* at least partly by improving the host’s cellular immunity, which means that this 20(R)-epimer is clinically more potent in the treatment of immune-mediated diseases (Wu et al., 2014). Interestingly, although 20(S)-Rg_3_ exhibits many potent activities in other clinical treatments, 20(R)-Rg_3_ seems to be a better epimer for the treatment of immune disorders based on the literature so far.

#### Antidiabetic Effects

To explore the difference of ginsenoside Rg_3_ epimers in antidiabetic effects, glucose-stimulated insulin secretion and phosphorylation of AMPK were examined in HIT-T15 and C2C12 myotubes, respectively. 5 mM of 20(S)-Rg_3_ enhanced insulin secretion to an extent comparable to 5 mM glipizide (58 and 61% increments), but 20(R)-Rg_3_ did not show any significant effect. In C2C12 myotubes, although both of these two epimers significantly promoted the phosphorylations of AMPK and acetyl-CoA carboxylase (ACC), 20(R)-Rg_3_ showed a little less effect. These results indicate that 20(S)-Rg_3_ might be a more potent anti-diabetic agent (Park et al., 2008). The antidiabetic activities of 20(S)-Rg_3_ have further been demonstrated, and the mechanisms on the treatment of diabetic renal damage and type 2 diabetes have been elucidated on this compound (Ju et al., 2012).

#### Anti-oxidant and Anti-photoaging Effects

20(R)-Rg_3_ has been found to be a more potent antioxidant compound than 20(S)-Rg_3_. 20(R)-Rg_3_ significantly inhibites oxidative stress in mice induced by cyclophosphamide based on the parameters of spleen and thymus, total antioxidant capacity, the activities of catalase, superoxidase dismutase, lysozyme, xanthine oxidase as well as the levels of malondialdehyde and nitric oxide (Wei et al., 2012b). Nevertheless, 20(S)-Rg_3_, but not 20(R)-Rg_3_, has been investigated to decrease intracellular reaction oxygen species (ROS) levels induced by UV-B in human keratinocyte HaCaT cells and human dermal fibroblast cells, respectively. Moreover, 20(S)-Rg_3_ exhibites UV-B-induced matrix metalloproteinase (MMP)-2 inhibition activities in HaCaT cells, while 20(R)-Rg_3_ does not (Lim et al., 2014). Although both 20(S)- and 20(R)-Rg_3_ may exhibit high anti-oxidant activities, the results are not contradictory. The experimental design of different animal models and cell lines may lead to different conclusions based on diverse therapeutic targets. Thus, further researches should be performed to elucidate the underlying mechanisms of these two stereoisomers.

### Stereospecific Effects of Other Saponin Stereoisomers

Except for ginsenoside Rg_3_, other stereoisomeric saponins have also been found to exhibit stereospecific pharmacological activities. For example, 20(S)-/20(R)-Rh_2_, the metabolites of 20(S)-/20(R)-Rg_3_, have also been studied on the stereospecific effects in their antitumor, anti-photoaging, anti-inflammatory, antioxidative, matrix metalloproteinase inhibitory and osteoclastgenesis inhibitory effects (Liu J. et al., 2009; Choi et al., 2013; Fatmawati et al., 2014; Oh et al., 2014). Compared with 20(R)-Rh_2_, 20(S)-Rh_2_ exhibits stronger anticancer effects on prostate cancer cells and human lung adenocarcinoma A549 cells (Liu et al., 2010a; Zhang et al., 2011). Moreover, the inhibitory effects of 20(S)-Rg_2_ on catecholamine secretion are slightly greater than those of 20(R)-Rg_2_ (Kudo et al., 1998).

These results reveal that the structural difference between saponin stereoisomers may lead to significant distinctions in pharmacological activities, and this phenomenon should be carefully considered in the future development of saponin-based therapeutics. Brief contrast of the pharmacological effects of stereoisomeric saponins with their corresponding stereoisomers is given in **Table 5**.

**Table 5:** Brief contrast of the pharmacological effects of ginsenoside Rg_2_ and Rh_2_ stereoisomers.

Name	Stereo-center	Pharmacological effects to be compared	Stereoisomer with more potent activities	Reference
Ginsenoside Rg_2_	20(S)/20(R)	Inhibition of catecholamine secretion	20(S)-Rg_2_	Kudo et al., 1998
Ginsenoside Rh_2_	20(S)/20(R)	Skin anti-photoaging activities	20(S)-Rh_2_	Oh et al., 2014
		Inhibition of osteoclastgenesis in RAW264 cells	20(R)-Rh_2_	Liu J. et al., 2009
		Inhibition of prostate cancer cells proliferation	20(S)-Rh_2_	Liu et al., 2010a
		Induction of apoptosis in human lungadenocarcinoma A549 cells	20(S)-Rh_2_	Zhang et al., 2011
		Anti-inflammatory, antioxidative and matrix metalloproteinase inhibitory activities in the LPS-stimulated murine RAW264.7 macrophage cells	20(R)-Rh_2_	Choi et al., 2013
		Inhibiting of erythroleukemia K562 cells proliferation	20(S)-Rh_2_	Xia et al., 2014
		Inhibition of aldose reductase	20(S)-Rh_2_	Fatmawati et al., 2014


### Pharmacological Effects of Other Stereoisomeric Saponins

Except for optical saponin epimers, geometric saponin epimers generated after the processing of *P. notoginseng* also exhibit high pharmacological activities, although the stereospecific effects have not been studied. For example, gingsenoside RK_1_ has been proved on its anti-platelet aggregation, anti-tumor, and vascular leakage blocking activities (Kim J.S. et al., 2012; Kim S.S. et al., 2014; Maeng et al., 2013). Gingsenoside Rg_5_, the geometric isomer of RK_1_, is shown to possess anti-inflammatory effects (Lee Y.Y. et al., 2013). Moreover, Rg_5_ and Rh_3_ can protect memory deficits induced by scopolamine (Kim E.-J. et al., 2013). Gingsenoside RK_3_ is capable to prevent apoptosis induced by hypoxia-reoxygenation in H9c2 cardiomyocytes (Sun et al., 2013).

The potent pharmacological activities of stereoisomeric saponins, which are mostly generated and acting as biomarkers in processed *P. notoginseng*, very well explain the pharmacological differences between the raw and processed herbs. Hence, the pharmacological behaviors of processed *P. notoginseng* are the combination of pharmacological activities of all the secondary saponins, which conforms to the theory of multi component multi targets of traditional Chinese medical drugs.

### Mechanism of Stereospecific Properties Between Saponin Stereoisomers

**FIGURE 3:**
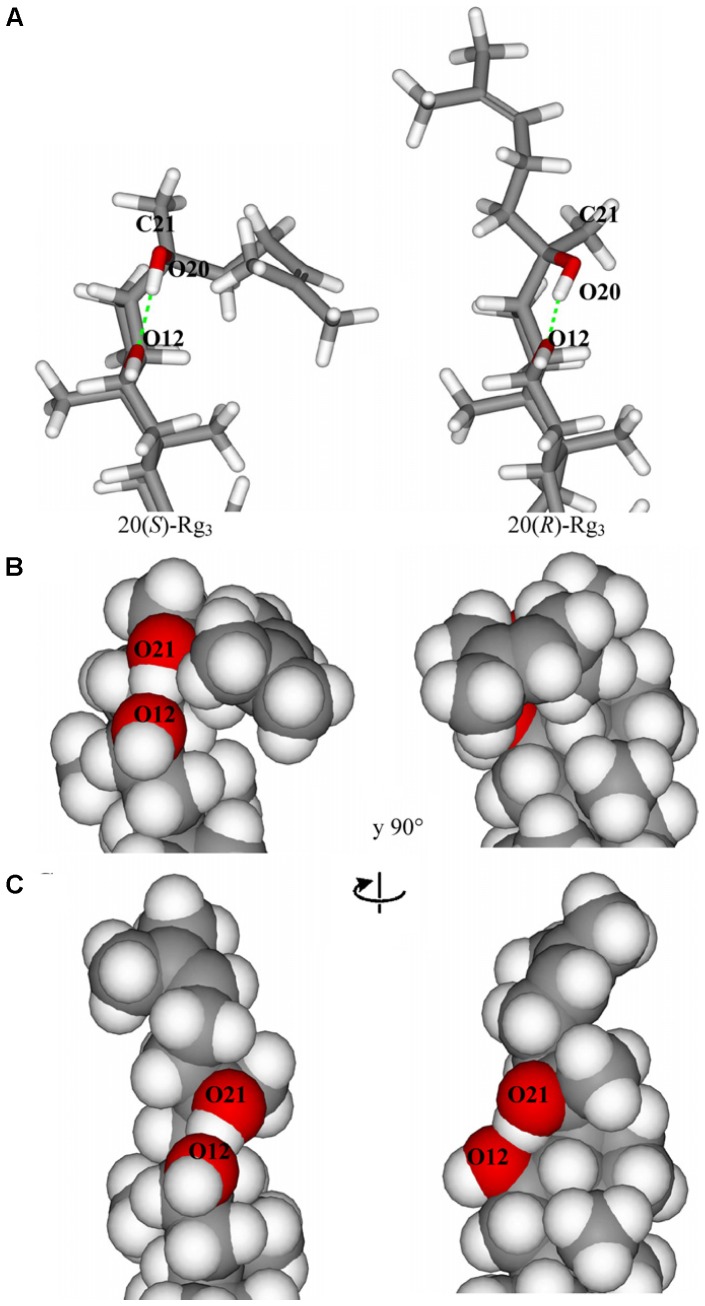
**(A)** Hydrogen bonds between HO20 and 012 in 20(S)- and 20(R)-Rg3. **(B)** Space filling representation of 20(S)-Rg_3_. **(C)** Space filling representation of 20(R)-Rg_3_ (adopted from Kang et al., 2005).

Based on the above reports, a diversity of pharmacological differences has been discovered in saponin stereoisomers. Unfortunately, very few studies have been investigated on the mechanism of this interesting phenomenon at a molecular level by far. Apparently, the position of the hydroxyl group on C-20 plays an important role on the different activities of stereoisomers of Rg_3_. The structure-activity relationship of 20(S)-/20(R)-Rg_3_ has been investigated on the determination of their difference in NMR spectroscopy and inhibition activities on Na^+^ channel current. The superimposition of 20 structures of 20(S)- and 20(R)-Rg_3_ indicates that the alkene chain of 20(R)-Rg_3_ is more flexible, however, that of 20(S)-Rg_3_ seems to be stable and tightly packed near the aglycon backbone. Moreover, the space filling representations of these two epimer (**Figure 3**) showed that the hydroxyl groups of HO12 and HO20 in 20(S)-Rg_3_ are less accessible to water compared with 20(R)-Rg_3_, while those of the latter epimer are found to be exposed to water. Taken together, the alignment of the 20(S)-Rg_3_ to receptor regions in Na^+^ channels is easier, and the hydrogen bonds occurred in 20(S)-Rg_3_ and receptor were more stable (Kang et al., 2005).

**FIGURE 4:**
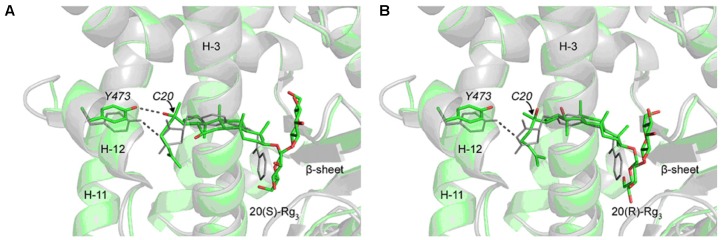
Docking structures of 20(S)-Rg_3_ and 20(R)-Rg3 in the LBD of PPAR *γ.* The ginsenosides and the side chain of Tyr473 in helix-12 (H-12) are shown in sticks. Crystal structure of rosiglitazone in PPAR γ (PDB:1ZGY) is superimposed and shown in gray for comparison. **(A)** The docked 20(S)-Rg_3_ adopts a position similar of rosiglitazone in the protein and the hydroxyl group at the C20 position interacts with the side chain of Tyr473 (dotted line). **(B)** The docked 20(R)-Rg_3_ occupies similar position as the 20(S)-Rg_3_ in the protein but only the methyl group at the C20 position points toward Tyr473 and does not form any effective hydrogen bond. The protein backbones are rendered as faded out ribbons. Hydrogen atoms are not shown for clarity. Both Rg_3_ stereoisomers were docked into the protein by using Autodock 4.0, while the receptor protein structure is directly obtained from the protein databank (adopted from Kwok et al., 2012).

The interaction of 20(S) and 20(R)-Rg_3_ with human serum albumin (HSA) has been investigated using surface-enhanced Raman scattering (SERS) and fluorescence spectroscopy. Results indicate that HSA is prone to bind to glucose ring upon the interaction with Rg_3_, with the aglycon part exposed outside. Thus when the combination of 20(S)- or 20(R)-Rg_3_-HAS complex with targeting binding site occurs, the stereoscopic spatial structures of these two epimers at C-20 may cause different binding affinities and biological activities (Zhang et al., 2014). Computational modeling has been utilized to elucidate the mechanism of stereospecific activities of 20(S)-Rg_3_ on peroxisome proliferator-activated receptor-gamma (PPARγ). It has been verified that Tyr473 in helix-12 is crucial to full agonistic activity of PPARγ. The docking results reveal that the hydroxyl group at C-20 of 20(S)-Rg_3_ interacts with Tyr473 via hydrogen bond. However, 20(R)-Rg_3_ is not able to interact with Tyr473 optimally due to its sterically strained binding pocket (**Figure 4**). It can be inferred that the biological activities difference of 20(S)- and 20(R)-Rg_3_ on PPARγ is due to their different binding affinities according to stereo-structures (Kwok et al., 2012).

## Pharmacokinetics

**FIGURE 5:**
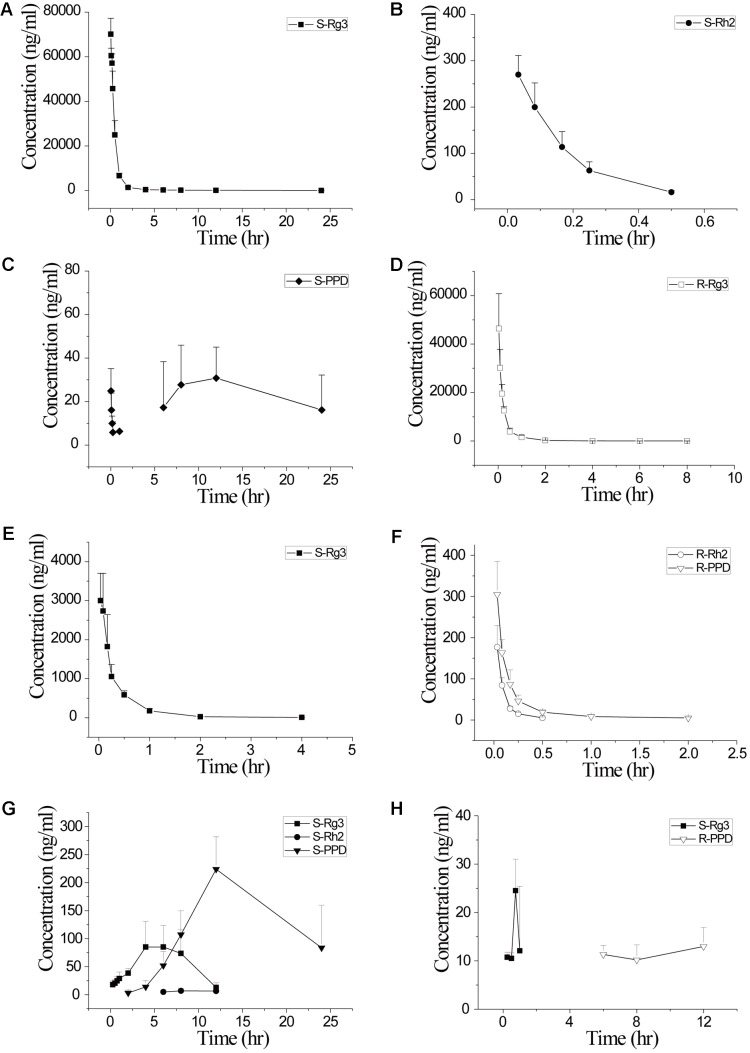
Plasma concentration-time curves of 20(S)- and 20(R)-ginsenoside Rg3 and their corresponding detectable chiral metabolites after iv administration of 20(S)-Rg3 (5 mg/kg dosage, **A–C**), iv administration of 20(R)-Rg3 (5 mg/kg dosage, **D–F**), ig administration of 20(S)-Rg3 (50 mg/kg dosage, **G**) and ig administration of 20(R)-Rg3 (50 mg/kg dosage, **H**) in rats (*n* = 6). Adopted from Peng et al. (2016a).

Saponin stereoisomers are proved to exhibit different pharmacokinetic characteristics including absorption, distribution, and metabolism. Bae et al. (2013) developed a LC-MS/MS method for simultaneous determination of Rg_3_ and Rh_2_ epimers, and the method was successfully applied to a pharmacokinetic study after oral administration of fermented ginseng extract in rats. 20(S)-epimers of both Rg_3_ and Rh_2_ showed significantly higher plasma concentrations and area under curve (AUC) values compared with their corresponding 20(R)-epimers. It can be inferred that 20(R)-epimers of Rg_3_ and Rh_2_ have lower membrane permeability and poorer absorption. Moreover, the absorption profiles of 20(S)-Rh_2_ was proved to be better than those of 20(R)-Rh_2_, partly because 20(R)-Rh_2_ performs more potent ABC-transporter-mediated efflux (Gu et al., 2010). Moreover, Bae et al. found that *Bacteroides* sp., *Eubacterium* sp., and *Bifidobacterium* sp. isolated from human fecal microflora can metabolize Rg_3_ to PPD via Rh_2_, however, *Fusobacterium* sp. is able to metabolize Rg_3_ to Rh_2_. The metabolism speed of 20(S)-Rg_3_ to its 20(S)-metabolites is 19-time higher than that of 20(R)-Rg_3_ (Bae et al., 2002). Our group has also found that after a same intravenous dosage of 5 mg/kg in rats, the AUC of 20(S)-Rg_3_ was about 2.3 times greater than that of 20(R)- Rg_3_. The half time of 20(S)-Rg_3_ was much longer and the clearance was lower (**Table 6**). A single direction chiral inversion of 20(R)-Rg_3_ to 20(S)-Rg_3_ was found in rats both after intravenous and intra-gastric administration. Both Rg_3_ epimers can undergo deglycosylated steps to their corresponding C20 chiral configurations of Rh_2_ and PPD, but the deglycosylation rates and patterns were different (**Figure 5**) (Peng et al., 2016a). The pharmacokinetic studies of stereospecific properties of ginsenoside stereoisomers may provide an experimental basis to explain the different activities of two stereoisomers.

**Table 6:** The main pharmacokinetic parameters of 20(S)-Rg_3_ and 20(R)-Rg_3_ in rat plasma after intravenous and intra-gastric administration.

Components	Administration Route	Dosage	C_max_	T_max_	AUC (h^∗^ng/ml)	*t*_1/2_(h)
20(S)-Rg_3_	i.v.	5 mg/kg	70096.2 ± 7204.6	0.033	39591.8 ± 1800.0	4.7 ± 1.5
20(R)-Rg_3_	i.v.	5 mg/kg	46439.2 ± 14365.5	0.033	11919.3 ± 1896.3	1.5 ± 0.4
20(S)-Rg_3_	i.g.	50 mg/kg	98.1 ± 40.5	5.7 ± 2.0	672.1 ± 308.1	2.2 ± 0.4


*Adopted from Peng et al. (2016a).*

## Discussions and Outlook

*Panax notoginseng* is a traditional Chinese medical drug rich in dammarane-type tetracyclic triterpenoid saponins. Some naturally existed major saponins will be deglycosylated or dehydrated to become secondary saponins during the heating processing of *P. notoginseng*. Researches on the chemical components of raw and processed notoginseng indicate that the transformation of compound basis is the basic reason for the changes in their pharmacological effects. A majority of these secondary saponins are stereoisomeric saponins which have been tentatively assigned to be “biomarkers” in processed notoginseng. Available investigations reveal that a diversity of distinctly different pharmacological activities, pharmacokinetic behaviors exhibits between two saponin stereoisomers.

The phenomenon of stereoisomerism has gained great attention on chemical drugs by many pharmaceutical enterprises. A very famous example is the disaster caused by thalidomide in 1960s in pharmaceutical history. 8000–12000 infants were born to be limb malformed owing to the exposure to this drug during the pregnancy period of their mothers (Melchert and List, 2007). The reason is that the structure of thalidomide is racemic, with R-(+) conformation exhibiting high anti-epileptic effect, while S-(-) conformation presenting strong teratogenic effect. However, the pharmacological difference in these two epimers has not been recognized at that time. Lessons have been learned from this tragedy that the separation and the study of drug stereoisomers are of great importance. With the development of the research on stereoisomeric drugs in recent years, the best four selling drug on the list of top ten global ranking list in 2006 were chiral drugs which were optically pure. However, stereoisomerism of natural compounds has not been studied extensively. Although stereoisomeric saponins have already attracted the interest of researchers, the investigations on the pharmacological differences have only been focused on ginsenoside Rg_3_ and Rh_2_, which are far below enough. More studies on pharmacological activities of minor saponin isomers produced in processed notoginseng, biological difference and pharmacokinetic behaviors in stereoisomeric saponins and the underlying mechanisms, and the preparation methods of stereoisomeric saponins need to be performed. Moreover, other cost-effective, efficient, and environmentally friendly microorganic and enzymatic hydrolysis methods for the preparation of optically pure saponins are worthy of further development and exploration. We believe that those researches on stereoisomeric saponins will be beneficial to structure-activity relationship, structure modification and new drug development in phytochemicals.

## Author Contributions

TZ and YD formulated the study concept and design of the paper. MP prepared the **Tables 2**–**6**. MP and YY acquired the data, draw the **Figure 1** and **Table 1**, and drafted the manuscript. JL guided the critical revision of the manuscript and provided important intellectual content. All authors reviewed the manuscript, agreed to all the contents, and agreed the submission.

## Conflict of Interest Statement

The authors declare that the research was conducted in the absence of any commercial or financial relationships that could be construed as a potential conflict of interest.
